# Intra-articular cervical facet joint corticosteroid injections in patients with increased peri-facet MRI STIR signal. A prospective, multi-center case series

**DOI:** 10.1016/j.inpm.2025.100646

**Published:** 2025-10-09

**Authors:** Joshua Levin, Kevin Barrette, Cyrus Ghaffari, Reza Ehsanian, Jayme Koltsov, Christina Giacomazzi, Nitin Prabhakar, Lisa Huynh, Matthew Smuck, William Summers, Byron Schneider

**Affiliations:** a Department of Orthopaedic Surgery, Stanford University, 450 Broadway St., Pavilion C; 4th Floor, MC 6342, Redwood City, CA, 94063, USA; b Department of Neurosurgery, Stanford University, 213 Quarry Rd., Palo Alto, CA, 94304, USA; c Orthopedic Surgery, Scripps, 2205 Vista Way Ste 210, Oceanside, CA, 92054, USA; d Pain Medicine, University of California, San Diego, 9400 Campus Point Dr., San Diego, CA, 92037, USA; e Anesthesiology and Critical Care Medicine, University of New Mexico, 2500 Marble Ave NE, Albuquerque, NM, 87106, USA; f Physical Medicine and Rehabilitation, Kaiser Permanente, 4650 Palm Ave, San Diego, CA, 92154, USA; g Physical Medicine and Rehabilitation, Kaiser Permanente, 1100 Veterans Blvd., Redwood City, CA, 94063, USA; h Physical Medicine and Rehabilitation, Vanderbilt University, 719 Thompson Lane, Suite 23108, Nashville, TN, 37204, USA

## Abstract

**Background:**

Intra-articular cervical facet joint corticosteroid injections are commonly performed, yet studies demonstrating benefit are limited.

**Purpose:**

To evaluate success rates of intra-articular cervical facet joint corticosteroid injections in patients with increased peri-facet edema as demonstrated by MRI with STIR sequences.

**Study design:**

Preliminary, prospective, multi-center case series.

**Patient sample:**

Thirty-three patients from three independent spine centers.

**Methods:**

Consecutive patients were enrolled with axial neck pain and peri-facet joint edema on MRI with STIR sequences when undergoing intra-articular cervical facet joint corticosteroid injections. Outcomes were prospectively collected at 2-4-weeks and at 3-months post-injection. The primary outcome was the proportion of patients with at least 50 % improvement in the numeric rating scale (NRS) pain score. Neck disability index (NDI) and global perception of change (GPC) were evaluated as secondary outcomes.

**Results:**

At 2-4-weeks post-injection, 64 % [95 %CI: 46–79 %] of the 28 patients with follow-up data met criteria for success (≥50 % improvement in NRS). 86 % [95 %CI: 69–94 %] reported that they were better or much better on the GPC, and mean NDI improved from 19.3 to 8.9. At 3-months post-injection, 35 % [95 %CI: 19–54 %] of the 26 patients with follow-up data met criteria for success, and 50 % [95 %CI: 32–68 %] reported that they were better or much better on the GPC. Mean NDI at 3-months was 11.0.

**Conclusions:**

Intra-articular cervical facet joint corticosteroid injections may provide short-term relief of neck pain in patients with peri-facet edema as demonstrated by MRI with STIR sequences. Intermediate-term results are less encouraging.

## 1 Introduction

Neck pain is the fourth leading cause of global disability behind low back pain, arthralgias, and depression [1]. Pain originating from the cervical facet joints is one of the leading etiologies of neck pain, with prevalence rates as high as 60 % after whiplash injury [2]. While conservative treatments are usually recommended prior to pursuing invasive procedures [3], evidence supporting the benefits of conservative management is limited [4].

Treatment of neck pain is challenging, and percutaneous radiofrequency neurotomy (RFN) of the medial branch nerves that innervate the facet joints is the only interventional treatment that has high-quality evidence demonstrating resolution of neck pain and elimination of the need for other healthcare resources for carefully selected patients with facet joint pain [5]. Efficacy from this procedure was supported in a prospective, randomized, placebo-controlled trial using strict patient selection criteria and proper technique [6]. In contrast, the evidence regarding effectiveness of intra-articular cervical facet joint corticosteroid injections is limited. One prospective, randomized, placebo-controlled study did not show benefit [7], but had notable limitations regarding patient selection and procedural factors. While the data supporting the use of intra-articular facet joint injections is limited, if shown to be an effective treatment in a certain population of patients, the procedure could potentially have clinical utility given the potential downsides of medial branch blocks/RFN, including logistical challenges associated with proper interpretation of medial branch block results, post-procedure weakness with dropped head syndrome [8], and post-RFN neuritis in as many as 1 in 5 patients [9].

Due to the ubiquitous nature of degenerative changes among both patients with neck pain and asymptomatics, abnormal findings on imaging studies, including x-ray, computed tomography (CT), magnetic resonance imaging (MRI), and bone scan, have not proven reliable at diagnosing patients with facet joint pain [3]. MRI with STIR (short tau inversion recovery) sequences effectively demonstrate edema, and may represent a method of diagnosing facet joint synovitis [10]. While corticosteroids may not be an effective treatment for all types of neck pain or facet arthropathy, patients with imaging evidence of facet joint synovitis may be more responsive to the anti-inflammatory effects from intra-articular corticosteroid injections. Outcomes have not been reported in this subset of neck pain patients.

The purpose of this preliminary, prospective, multi-site, observational study was to gather outcomes data on patients who would appear to be the most likely to benefit from intra-articular cervical facet joint corticosteroid injections. Our hypothesis was that these patients may benefit from these injections, and encouraging results would provide motivation to pursue further high-quality randomized study.

## 2 Methods

**Fig. 1:**
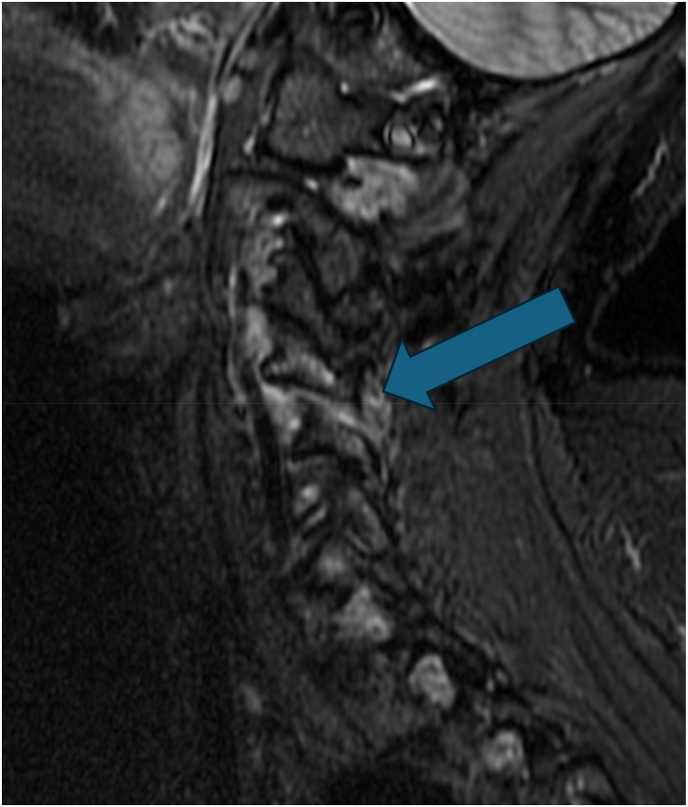
Sagittal STIR MRI with C4-5 peri-facet joint edema (arrow).

The study was approved by institutional review boards at three separate institutions (IRB #45400, 181852, and 22–7894), and the study was conducted according to the Declaration of Helsinki. Consecutive patients were prospectively enrolled at three different sites, and informed consent to participate in the study was obtained from all patients. Inclusion criteria consisted of patients ≥18 years old with unilateral or bilateral axial neck and/or shoulder girdle/peri-scapular pain of at least 4 weeks duration with a 7-day average NRS pain score ≥4/10 at baseline. Patients were required to have an MRI with STIR images demonstrating increased peri-facet signal as determined by the treating physiatrist at one or more facet joints correlating with the side and location of the patient's symptoms (Fig. 1). Patients with a history of posterior spine surgery and those with a grade 2 or greater spondylolisthesis at the involved or adjacent segments were excluded.

Consecutive patients who met the criteria and planned on undergoing intra-articular (IA) cervical facet joint corticosteroid injections as part of their routine clinical care were enrolled in the study. All joints that demonstrated increased peri-facet joint signal were injected in each patient. The NRS pain score and neck disability index (NDI) were collected at baseline, 2-4-weeks post-injection, and at 3-months post-injection. Global Perception of Change (GPC) was collected at 2-4-weeks post-injection and at 3-months post-injection. As the injections were part of the usual care for each patient, there were no controls on other treatments, such as medications or physical therapy.

Injections were performed according to International Pain and Spine Intervention Society (IPSIS) Practice Guidelines [11] for cervical facet joint intra-articular access by fellowship trained interventional spine physiatrists and/or their fellows under direct supervision. After proper needle placement was confirmed using bi-planar fluoroscopic views and production of a facet joint arthrogram under real-time fluoroscopy with contrast, 1 cc of DepoMedrol 20 mg mixed with 1 % lidocaine was injected into each facet joint (a smaller volume was injected if the capacity of the joint was met prior to the injection of 1 cc) (Fig. 2a, Fig. 2b, Fig. 2c, Fig. 2da–d).

**Fig. 2a:**
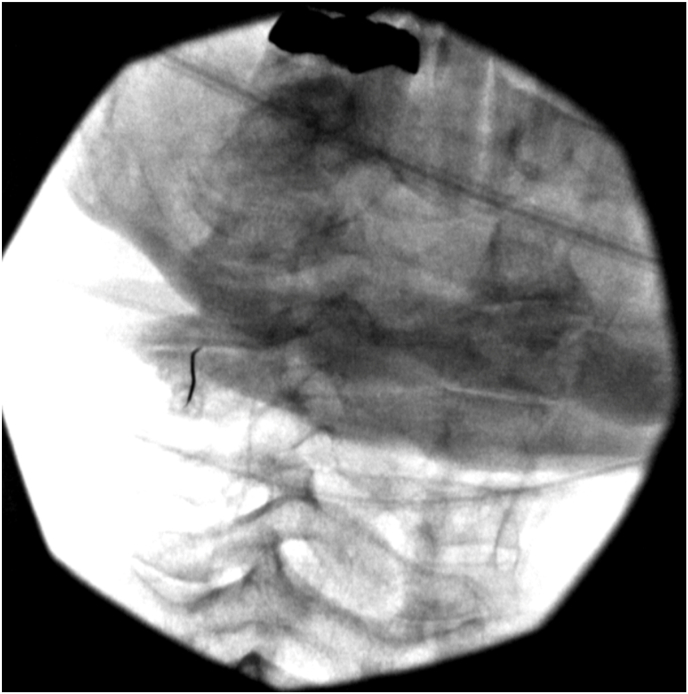
Pre-contrast AP image with needle in the left C4-5 facet joint.

**Fig. 2b:**
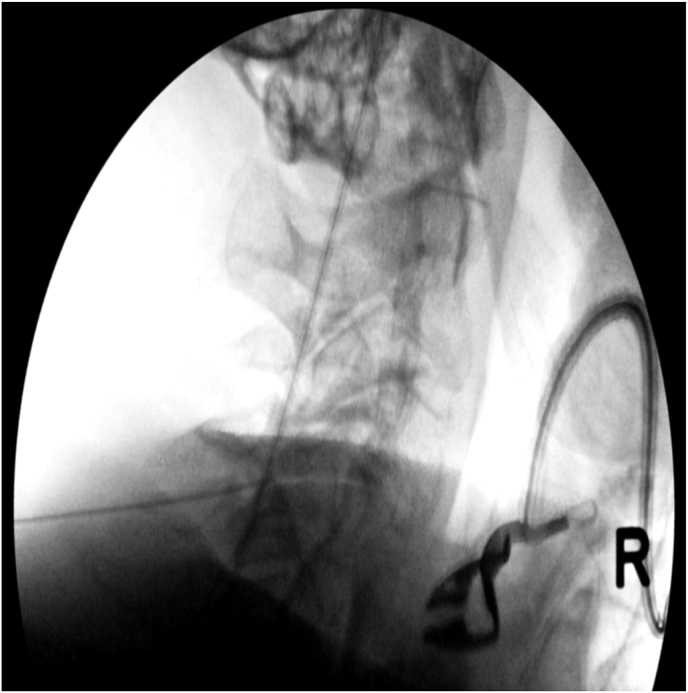
Pre-contrast lateral image with needle in the left C4-5 facet joint.

**Fig. 2c:**
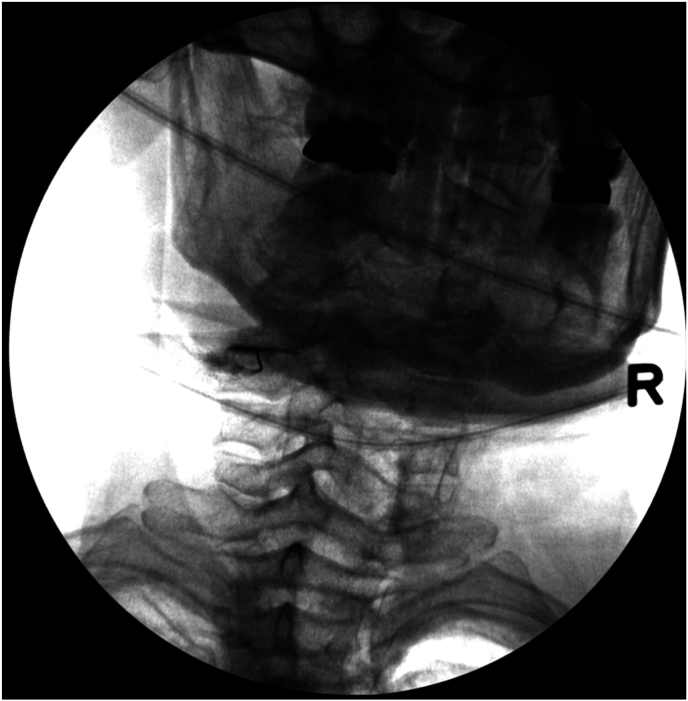
Post-contrast AP image demonstrating a left C4-5 facet joint arthrogram.

**Fig. 2d:**
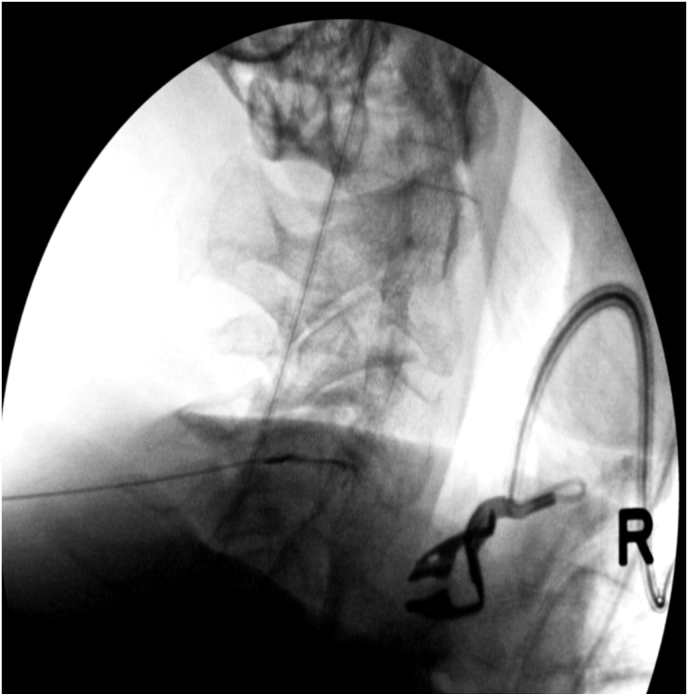
Post-contrast lateral image demonstrating a left C4-5 facet joint arthrogram.

## 3 Statistical analysis

The primary outcome was categorical response to the injection, with successful outcome defined as ≥50 % improvement from baseline NRS pain and no additional procedures of the facet joints (such as medial branch blocks or RFN) at 2-4-weeks and at 3-months post-injection. Patients having RFN were defined as treatment failures. Secondary outcomes included the proportion of patients who reported that they were better or much better on the GPC and change in raw pain and NDI scores from baseline. Success rates and the percent of patients reporting that they were better or much better are described as frequencies and percentages with Wilson 95 % confidence intervals. Additionally, for success rates and the GPC, three sensitivity analyses were undertaken: the first assuming patients with missing NRS pain scores at 2-4-weeks and/or 3-months all failed treatment, the second assuming that patients with missing NRS pain scores at 2-4-weeks and/or 3 months all succeeded, and the third assuming no change between 2-4-weeks and/or 3 months (carrying over any scores to replace missing data). The change in raw NRS pain scores and NDI scores were assessed with Mann-Whitney U tests with Bonferroni correction for multiple time points and descriptive statistics are presented as means and standard deviations. All analyses were performed with SAS version 9.4 (Cary, NC, USA) with a two-sided level of significance of α = 0.05.

An a-priori power calculation demonstrated that 30 patients would provide at least 90 % power to detect improvements of the minimum clinically important difference (MCID) (conservatively estimated at 1.5 points [12]) or larger in NRS pain scores via Bonferroni-corrected paired t-tests with a two-sided level of significance of 0.05. This calculation assumes the typical variability in NRS pain scores observed in similar populations [13], and includes an additional 15 % cushion in the event of loss to follow up. This sample size provides over 80 % power to determine the percentage of patients having ≥50 % improvement from baseline NRS pain scores to within a confidence interval of ±15 %.

Patients who subsequently underwent RFN were excluded from analysis for time points thereafter so as not to conflate response to RFN as a treatment response to the IA corticosteroid injection.

## 4 Results

Thirty-three consecutively consenting patients were enrolled with a mean age of 62 ± 8 years and a median duration of symptoms of 11 months [interquartile range [IQR]: 5, 18]. They received intra-articular cervical facet joint injections between July 19, 2018 and November 16, 2022 at 3 separate institutions (Table 1). Twenty-nine patients (87.9 %) had one joint injected unilaterally, three patients had two joints injected unilaterally (9.1 %), and one patient had one joint injected bilaterally (3.0 %), for a total of 37 joints. The most commonly injected joints were C3-4 and C4-5 (Table 2).

**Table 1:** Baseline demographics and clinical information. Data is presented as n (%) for categorical variables and means ± standard deviation or median [IQR] for continuous variables.

Demographics	Descriptive Statistics
Female	19 (57.6 %)
Age (years)	62.3 ± 7.6
BMI (kg/m^2^)	26.4 ± 4.3
Duration of pain (months)	11.0 (IQR 4.5, 17.5)

**Table 2:** Number of injections performed at each level, presented as n (%) (4 patients had two joints injected; total number of injections = 37).

	n (%)
**Laterality**	
Left side	16 (48.5 %)
Right	16 (48.5 %)
Bilateral	1 (3.0 %)
**Level**	
C2-3	5 (13.5 %)
C3-4	16 (43.2 %)
C4-5	15 (40.5 %)
C5-6	0 (0.0 %)
C6-7	0 (0.0 %)
C7-T1	1 (2.7 %)

**Fig. 3:**
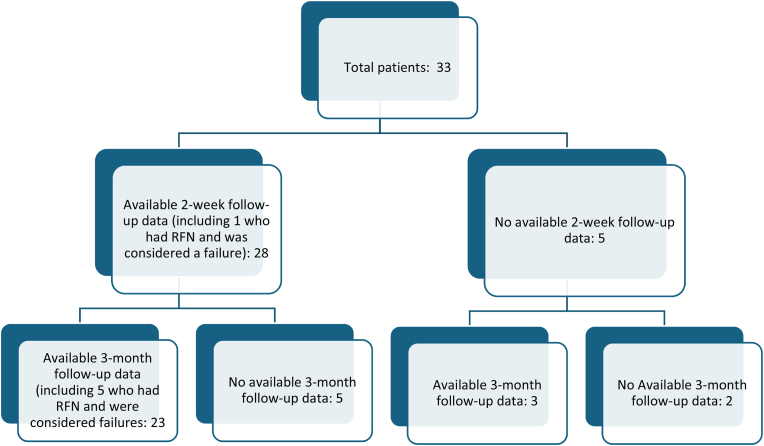
Available follow-up data CONSORT diagram.

**Fig. 4:**
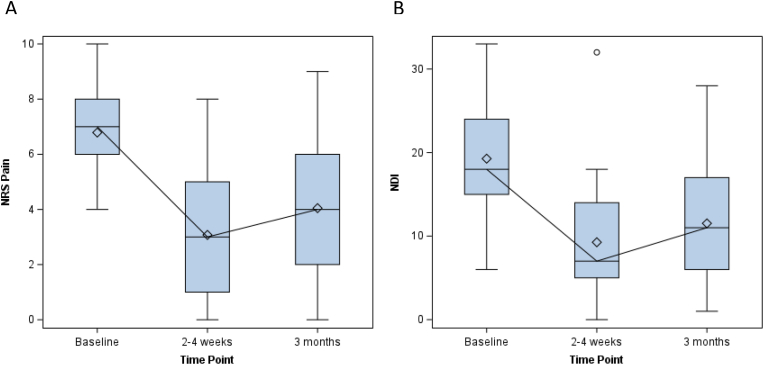
(A) NRS pain scores and (B) NDI scores by time point. Central lines indicate medians and central dots indicate means. Box widths show the IQR, and whiskers show 1.5xIQR.

In the 28 patients with available follow-up data at 2-4-weeks (5 patients were unable to be contacted), and the 26 patients with available follow-up data at 3-months (5 patients were unable to be contacted and 2 patients refused to complete the post-injection data collection sheet) (Fig. 3), the success rates based on NRS pain score and not having any RFN post-injection were 64.3 (95 % CI: 45.8, 79.3) % at 2-4-weeks and 34.6 (19.4, 53.8) % at 3-months (Table 3). Failures included one patient (3.0 %) who had an RFN by 2-4-weeks and 5 (15.2 %) who had an RFN by 3-months. Mean NRS pain score was 6.8 (S.D. = 1.5) at baseline and decreased to 3.1 (S.D. = 2.1) and 3.9 (S.D. = 3.0) at 2-4-weeks and 3-months, respectively (Fig. 4). The sensitivity analyses ranged from 54.6 (38.0, 70.2) % to 69.7 (52.7, 82.6) % success at 2-4-weeks and 27.3 (15.1, 44.2) % to 48.5 (32.5, 64.8) % at 3 months (Table 3).

**Table 3:** Frequency and percentages of patients meeting improvement thresholds (50 %) in NRS pain score and Better/Much better on Global Perception of Change (GPE).

Outcome	Time Point	Original Data	Missing Coded as Fail	Missing Coded as Success	Missing Replaced from Other Timepoints
**NRS Success**	2–4 Weeks	64.3 (45.8, 79.3) %	54.6 (38.0, 70.2) %	69.7 (52.7, 82.6) %	61.3 (43.8, 76.3) %
	3 Months	34.6 (19.4, 53.8) %	27.3 (15.1, 44.2) %	48.5 (32.5, 64.8) %	35.5 (21.1, 53.1) %
**GPE Success**	2–4 Weeks	85.7 (68.5, 94.3) %	72.7 (55.8, 84.9) %	87.8 (72.7, 95.2) %	80.7 (63.7, 90.8) %
	3 Months	50.0 (32.1, 67.9) %	39.4 (24.7, 56.3) %	60.6 (43.7, 75.3) %	58.1 (40.8, 73.6) %

Mean NDI was 19.3 (S.D = 6.6) at baseline and decreased to 8.9 (S.D. = 7.0) and 11.0 (S.D. = 7.9) at 2-4-weeks and 3-months, respectively (Fig. 4).

On the Global Perception of Change, 85.7 (68.5, 94.3) % of patients at 2-4-weeks and 50.0 (32.1, 67.9 %) % at 3-months rated themselves as improved or much improved (patients who had RFNs were rated as not improved). In sensitivity analyses, this ranged from 72.7 (55.8, 84.9) % to 87.8 (72.7, 95.2) % at 2-4-weeks and 39.4 (24.8, 56.3) % to 60.6 (43.7, 75.3) % at 3-months (Table 3).

Excluding the one patient who underwent RFN by 2-4-weeks, the median NRS pain score of the remaining 27 patients decreased by 4 (IQR: 2, 5) points relative to baseline (p < 0.001) and the median NDI score decreased by 10 (4, 16) points relative to baseline (p < 0.001). Of the 21 patients with complete data at 3 months (excluding the 5 patients who underwent RFN), median NRS pain score decreased by 2 (IQR: 1, 5) points relative to baseline (p = 0.005) and median NDI decreased by 6 (IQR: 2, 13) points relative to baseline (p = 0.003).

## 5 Discussion

Here we present the first prospective cohort of outcomes in patients with cervical facet joint pain identified via peri-articular STIR signal on MRI who received a fluoroscopic guided IA facet joint corticosteroid injection. Since MRI with STIR signal may be able to identify cervical facet joint synovitis [10], we hypothesized that intra-articular facet joint corticosteroid injections are more likely to be helpful in these patients given their potent anti-inflammatory effects. The strong clinical effectiveness of cervical medial branch RFN is largely dependent on patient selection via a physiologic marker that predicts treatment success, specifically pain relief occurring after diagnostic medial branch blocks [14]. Part of our hypothesis was that this simple radiographic biomarker may be able to identify a subset of patients who respond to IA facet joint corticosteroid injections, even though this treatment has not demonstrated similar benefits relative to RFN in the general neck pain population [7]. Prior studies showed mixed results from facet joint injections in patients with abnormal uptake on bone scan [[15], [16], [17]], and correlation of abnormal bone scan findings with MRI findings is low [18]. Therefore, we sought to determine if peri-facet edema on MRI with STIR could select patients who would benefit from intra-articular corticosteroid injections.

Although our study did show reasonable success at 2-4-weeks post-injection, 64 % success, or 55 % in the worst-case scenario analysis, the success rate is similar to those seen in other uncontrolled interventional spine procedures [[19], [20], [21]], including many that were subsequently shown to lack efficacy in placebo-controlled trials [[22], [23], [24]]. The improvement in mean/median NRS pain score from 7 at baseline to 4 at 3-months did reach the minimum clinically important difference (MCID) in the treatment of neck pain [25]. However, by 3-months, the success rate in the primary outcome was nearly cut in half, down from 64 % at 2-4-weeks, to 35 %, or 27 % in the worst-case scenario analysis, in this uncontrolled study, suggesting primarily short-term benefits. While this treatment may benefit some patients, based on these results, we do not recommend its routine use in patients with increased peri-facet STIR signal and instead reserve its use in cases where more effective RFN is not available, such as in patients with contraindications to RFN, or at C7-T1 where anatomic studies have yet to isolate the location of the target nerves for RFN. Importantly, our results are similar to those observed in studies of corticosteroid injections into other synovial joints, with reasonably good initial pain relief, but waning relief in the medium and long term [26,27].

An interesting finding in our study was that the most commonly affected joints in our population were the C3-4 and C4-5 joints, accounting for 84 % of the joints with peri-facet edema. This differs from prior studies, primarily on whiplash or other injury patients, that selected patients based on diagnostic medial branch blocks, in which the most commonly affected joints were C2-3 and C5-6 [5]. This may suggest a different underlying pathology of injury-related cervical facet joint arthrosis as diagnosed by medial branch blocks compared to cervical facet joint synovitis/peri-facet edema.

Our study has limitations. First, there was no control group. Patients received the injections as part of their usual care, and there was no control for co-interventions. It is possible that some of the successes were due to other interventions (physical therapy, medications, alternative treatments, etc.) or to natural history alone. Second, at 2-4-weeks, our loss to follow-up rate was 15 %, and at 3-months it was 21 %. In evaluating our data, we performed both a worst-case and a best-case scenario analysis. The authors feel that a worst-case scenario analysis, especially in an uncontrolled study, is the more appropriate way of interpreting the data.

## 6 Conclusion

Intra-articular cervical facet joint corticosteroid injections may provide immediate benefit in some patients with peri-facet edema demonstrated by MRI with STIR sequences. Intermediate-term relief at 3-months is less encouraging, although may be beneficial in some patients.

## Declaration of competing interest

The authors declare the following financial interests/personal relationships which may be considered as potential competing interests: Joshua Levin reports a relationship with State Farm Insurance Companies that includes: consulting or advisory and paid expert testimony. Joshua Levin reports a relationship with Allstate Insurance that includes: consulting or advisory. Joshua Levin reports a relationship with Liberty Mutual Insurance Company that includes: consulting or advisory. Joshua Levin reports a relationship with GEICO that includes: consulting or advisory. Joshua Levin reports a relationship with Amazon that includes: consulting or advisory. Kevin Barrette reports a relationship with 10.13039/100008497Boston Scientific Corporation that includes: speaking and lecture fees. Matthew Smuck reports a relationship with Boston Scientific Corporation that includes: funding grants. Matthew Smuck reports a relationship with Axial Consulting Group that includes: consulting or advisory. Byron Schneider reports a relationship with State Farm Insurance Companies that includes: consulting or advisory. Byron Schneider reports a relationship with North American Spine Society that includes: board membership. Byron Schneider reports a relationship with International Pain and Spine Intervention Society that includes: board membership. Lisa Huynh reports a relationship with International Pain and Spine Intervention Society that includes: board membership. co-author serves an editorial role for the journal Interventional Pain Medicine - L.H. - Given her role as an editorial board member, she had no involvement in the peer review of this article and had no access to information regarding its peer review. Full responsibility for the editorial process for this article was delegated to another journal editor. co-author serves an editorial role for the journal Interventional Pain Medicine - M.S. - Given his role as an editorial board member, he had no involvement in the peer review of this article and had no access to information regarding its peer review. Full responsibility for the editorial process for this article was delegated to another journal editor. If there are other authors, they declare that they have no known competing financial interests or personal relationships that could have appeared to influence the work reported in this paper.
